# Expression of Concern: Elevated microRNA-145 inhibits the development of oral squamous cell carcinoma through inactivating ERK/MAPK signaling pathway by down-regulating HOXA1

**DOI:** 10.1042/BSR-2018-2214_EOC

**Published:** 2022-09-26

**Keywords:** Extracellular-signal-regulated kinase/mitogen activated protein kinase, Homeobox A1, MicroRNA-145, Oral squamous cell carcinoma

The Editorial Office has been made aware of potential issues surrounding the scientific validity of this paper hence has issued an Expression of Concern to notify readers whilst the Editorial Office investigates. A duplicated, modified region has been identified between the miR-145 mimic and siHOXA1 panels of Figure 4C, as well as concerns raised regarding the western blots in Figures 6 and 7.

